# Antiviral activity of berberine

**DOI:** 10.1007/s00705-020-04706-3

**Published:** 2020-06-28

**Authors:** Alicja Warowicka, Robert Nawrot, Anna Goździcka-Józefiak

**Affiliations:** 1 Department of Animal Physiology and Developmental Biology, Institute of Experimental Biology, Faculty of Biology, Adam Mickiewicz University, Poznań, Poland; 2 NanoBioMedical Centre, Adam Mickiewicz University, Poznań, Poland; 3 Molecular Virology Research Unit, Institute of Experimental Biology, Faculty of Biology, Adam Mickiewicz University, Poznań, Poland

## Abstract

Plants are a rich source of new antiviral, pharmacologically active agents. The naturally occurring plant alkaloid berberine (BBR) is one of the phytochemicals with a broad range of biological activity, including anticancer, anti-inflammatory and antiviral activity. BBR targets different steps in the viral life cycle and is thus a good candidate for use in novel antiviral drugs and therapies. It has been shown that BBR reduces virus replication and targets specific interactions between the virus and its host. BBR intercalates into DNA and inhibits DNA synthesis and reverse transcriptase activity. It inhibits replication of herpes simplex virus (HSV), human cytomegalovirus (HCMV), human papillomavirus (HPV), and human immunodeficiency virus (HIV). This isoquinoline alkaloid has the ability to regulate the MEK-ERK, AMPK/mTOR, and NF-κB signaling pathways, which are necessary for viral replication. Furthermore, it has been reported that BBR supports the host immune response, thus leading to viral clearance. In this short review, we focus on the most recent studies on the antiviral properties of berberine and its derivatives, which might be promising agents to be considered in future studies in the fight against the current pandemic SARS-CoV-2, the virus that causes COVID-19.

## Introduction

**Fig. 1:**
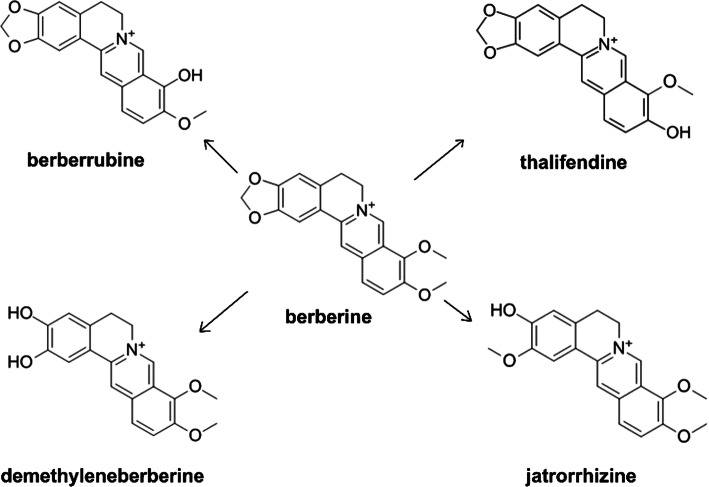
Berberine and its derivatives

Berberine (BBR) is a natural isoquinoline alkaloid with low toxicity. It is present in several medicinal plants, such as *Berberis vulgaris*, *Coptis chinensis, Hydrastis canadensis*, *Coptidis rhizoma*, *Xanthoriza simplicissima, Phellodendron amurense*, and *Chelidonium majus*. Berberine exhibits unusual biochemical and pharmacological activities, including antidiabetic [1], hypolipidemic [2], antihypertensive [3], anti-inflammatory [4], antidiarrheal [5], hepatoprotective [6], antidepressant [7], anticancer [8], antibacterial [9], and antiviral [10] properties. BBR is capable of penetrating all cell lines, but the cumulative concentration is the highest in Hep G-2 cells [11]. It can cross the blood-brain barrier when it is administrated systematically, and it has a protective effect on the central nervous system [12]. Due to its various properties, BBR is widely used as a dietary supplement. It has low toxicity and is well tolerated by the human body. However, high doses of BBR can cause gastrointestinal side-effects. In liver cells, BBR is metabolized with the participation of cytochrome P450 1A2 (CYP1A2), cytochrome P450 3A4 (3A4), cytochrome P450 2D6 (2D6), and UDP glucuronosyltransferases. In phase I, it is metabolized by demethylation, and in phase II, by glucuronidation. Its metabolites are berberrubine, demethylene-berberine, jatrorrhizine, thalifendine, and its glucuronidated derivatives [Fig. 1] [13–15]. BBR is administered by oral gavage, but its bioavailability is low. Currently, nanomaterials can be applied as an effective drug delivery system providing time-controlled and site-specific delivery of the loaded drug. Conjugation of BBR with liposomes or micelles allows its bioavailability to be improved. In recent years, empirical evidence has shown that this bioactive plant alkaloid possesses strong antiviral activity against different viruses. The antiviral activity of BBR against herpesviruses, influenza virus, and respiratory syncytial viruses has been scientifically documented. Its potential activity against SARS-CoV and other coronaviruses, as discussed below, also raises the question if it could be effective against the novel pandemic SARS-CoV-2 coronavirus, which is currently an overwhelming public-health problem worldwide. The search for novel therapeutics against this virus and the symptoms of its disease, COVID-19, are of the highest importance.

## Mode of the antiviral activity of berberine

Viruses modulate and utilize many host cellular processes for their replication. Virus infection can result in changes in cellular metabolism and signaling that facilitate viral replication. These processes involve different molecules, including nuclear factor kappa-light-chain-enhancer of activated B cells (NF-κB) and mitogen-activated protein kinases (MAPKs). NF-κB is a DNA-binding protein that is required in the transcription of various genes involved in controlling cellular processes, in particular, inflammatory and immune responses. The modulation of NF-κB pathway by BBR is one of the mechanisms that inhibits virus infection. BBR downregulates virus-induced NF-κB activation and blocks degradation of the endogenous NF-κB inhibitor IκBα. MAPKs are involved in the regulation of key cellular signaling pathways, such as apoptosis, differentiation, proliferation, and immune responses. MAPKs play an essential role in infection and cellular stress. Moreover, they are known to promote survival and generation of progeny virions. Four subgroups of MAPKs have been identified, namely, extracellular-signal-regulated kinase (ERK) 1 and 2, ERK5, isoforms of p38 protein (p38), and c-Jun N-terminal kinases (JNK) [16]. The JNK and p38 pathways play a key role in inflammation and tissue homeostasis. MAPKs are responsible for the phosphorylation of serine and threonine in many proteins. Phosphorylation plays a significant role in protein interactions, protein folding, and activation and deactivation of enzymes. ERK kinases phosphorylate multiple substrates, such as c-Fos and Elk 1, which are involved in the regulation of cell cycle progression and survival. Protein phosphorylation also plays an essential role in the infection cycle of many viruses [17]. A number of viruses induce or inhibit the phosphorylation of cellular proteins at all levels of signal transmission pathways from the plasma membrane to the nucleus. Phosphorylation can affect a viral protein’s stability, activity, interaction with other cellular and viral proteins, and infectivity. Viruses such as Epstein-Barr virus (EBV) [18], hepatitis C virus (HCV) [19], and coronavirus type 2 [20] activate the phosphorylation of MAPK. Changes in the phosphorylation of proteins have been observed during viral replication. For example, the human immunodeficiency virus (HIV) protein p6 is phosphorylated at a specific site (Thr 23) by MAPK and ERK-2. Mutational analysis has demonstrated that Thr 23 is important for the infectivity, maturation, and budding of viral particles [21]. Phosphorylation of the coat protein (CP) of RNA viruses can significantly affect CP-RNA interactions, the stability of viral particles, and the viral infection processes [22, 23]. Moreover, many viruses use phosphorylation of proteins to modulate signaling in order to prevent apoptosis and promote cellular survival and proliferation [24].

An example is chikungunya virus (CHIKV). It is transmitted to humans mainly by infected mosquitoes, specifically *Aedes aegypti* and *Aedes albopictus*. CHIKV causes fever, joint swelling, headache, and severe persistent muscle and joint pain in humans. CHIKV belongs to the family *Togaviridae* and has a positive-sense RNA genome. Host macrophages are the major cellular reservoirs of CHIKV during infection, and the production of the cytokines TNF, IL-1β, and IL-6, IL-8 may be associated with viral pathogenesis. During CHIKV infection, the major mitogen-activated protein kinase signaling pathways, p38, JNK, and ERK are activated. Activation of ERK and JNK kinases are essential for generation of CHIKV progeny virions. The specific molecular mechanism of CHIKV-induced MAPKs is not known. It has been shown that CHIKV can modulate the phosphorylation of p38 and JNK. Recently, it was demonstrated that the CHIKV nsP2 protein interacts with p38 and JNK in host macrophages [25]. In addition, JNK modulates the replication of certain viruses. For example, replication of HSV, HIV, and rotaviruses is suppressed by JNK inhibition, while the replication of influenza virus is increased. CHIKV induces MAPK activation as well as expression of viral nonstructural and structural proteins. Varghese et al. showed that BBR significantly reduces CHIKV-induced activation of MAPKs. The P38, ERK and JNK signaling pathways are strongly inhibited upon treatment with BBR, which especially targets the ERK signaling pathway, resulting in a marked reduction in particle formations. The reduction in viral protein expression after BBR treatment is probably a consequence of a decrease in virus-induced signaling. BBR treatment does not inhibit virus entry or the enzymatic activity of the viral replicase [26]. Furthermore, CHIKV induces prosurvival signal cascades such as PI3K-AKT and autophagy [27]. It has also been reported that BBR significantly reduces phosphorylation of p38 MAPK during respiratory syncytial virus (RSV) infection [28]. p38 MAPK is induced in the early stage of RSV infection [29]. Inhibition of the activity of p38 MAPK by BBR has also been observed during hepatitis B virus (HBV) infection. HBV is a member of the family *Hepadnaviridae*. Its virion contains a partially double-stranded relaxed circular DNA (rcDNA) genome, which, in the nucleus of an infected cell, is converted to covalently closed circular DNA (cccDNA). p38 MAPK plays a central role in the maintenance of HBV cccDNA in infected cells [30]. The cccDNA acts as a template for RNA synthesis, including mRNAs and pregenomic RNAs (pgRNAs). During the life cycle of HBV, pgRNA is converted to partially double-stranded rcDNA in the capsid by reverse transcriptase (RT). Inhibition of the activity of p38 MAPK is positively correlated with the suppression of HBV surface antigen (HBsAg) production, HBV e-antigen (HBeAg) secretion, and inhibition of HBV replication. It has been reported that BBR also intercalates into DNA, inhibiting DNA synthesis and reverse transcriptase activity [31–34]. HBV infection is associated with a broad spectrum of liver diseases, including acute hepatitis, chronic hepatitis, cirrhosis, and hepatocellular carcinoma. About a million deaths globally are caused by HBV-related diseases each year, with more than a million people suffering from chronic hepatitis B worldwide [35]. The ability of BBR to inhibit MAPK might make it a potential candidate for a new antiviral agent against HBV infection. p38 MAPK has been suggested as a possible target for anti-HBV therapy. Kim et al. showed that biphenyl amides act as p38 MAPK inhibitors and suppress HBsAg secretion [36]. Curcumin, another well-known alkaloid, decreases the level of HBsAg and reduces the number of cccDNA copies, thus inhibiting HBV replication and expression. Moreover, this natural plant compound reduces the acetylation level of cccDNA-bound histone H3 and H4 [37]. The current anti-HBV therapy options have disadvantages. These include high cost and cumulative toxicity, which results in bone disease and renal injury. Additionally, the therapy is limited to patients with viremia and elevated alanine aminotransferase (ALT) or fibrosis [38], and the MAPK pathway appears to be activated by other viruses, such as HCV [39], dengue virus [40], coronavirus [41], Venezuelan equine encephalitis virus (VEEV) [42], and enterovirus 71 (EV71) [43].

Another strategy that plays an important role in the replication of some viruses is autophagy. Autophagy is an intracellular degradation process that helps to maintain cellular homeostasis under both normal and stress conditions. It is a dynamic process starting with autophagosome formation, followed by fusion with lysosomes and degradation of the enclosed cargo. Autophagy is also an antiviral mechanism that selectively degrades viral components or viral particles inside lysosomes [44]. One example of a virus that exploits autophagy is the enterovirus EV71, a member of the family *Picornaviridae.* Picornaviruses are nonenveloped viruses with a positive-stranded RNA genome of 7.5 kb that contains a single open reading frame (ORF) flanked by 5’ and 3’ untranslated regions. This RNA serves as a template for translation of the viral polyprotein and for the amplification of the viral genome. Viral replication takes place on intracellular membranous structures and is dependent on autophagy. Enteroviruses induce autophagy to promote their own replication [45]. The mechanism responsible for this pro-viral function of autophagy is unknown. EV disrupts autophagosome-lysosome fusion, which allows viral RNA and proteins to escape degradation and consequently facilitates viral replication by providing membranes for replication, leading to pathogenesis in the host. EVs are associated with neurological disorders, cardiovascular damage, and metabolic disease. Recently, it has been reported that BBR can inhibit virus-induced autophagy and reduce viral RNA and protein synthesis [46]. BBR inhibits EV71-induced autophagy by affecting JNK, PI3KIII and AKT signaling. For example, BBR treatment increases AKT phosphorylation and reduces JNK and PI3KIII phosphorylation. JNK signaling is involved in autophagy, and its inhibition can inhibit this process. BBR also inhibits the MEK/ERK signaling pathway, which is important for mediating innate immunity to viral infections and plays a significant role in EV71 replication and pathogenesis [43, 46]. This suggests that BBR acts by inhibiting MAPK signaling and is a potential agent for the treatment of enterovirus infection, especially since there are no other effective and licensed drugs available.

## Activity of berberine against different viruses

### Activity of BBR against viruses of the family *Flaviviridae*

In the past decades, HCV has become a major public-health problem globally. At present, there is no effective vaccine against this virus. HCV is an enveloped, positive-strand RNA virus belonging to the family *Flaviviridae*. HCV infection is associated with a broad spectrum of liver diseases, including cirrhosis and hepatocellular carcinoma (HCC). The viral proteins E1 and E2 mediate entry of the virus into cells, which is a key step in its life cycle. The HCV E2 is primarily responsible for preventing premature membrane fusion, as well as stabilizing attachment of the virus to cells. Hong et al. have shown that BBR suppresses hepatitis C virus replication by targeting the viral E2 glycoprotein, specifically blocking HCV attachment and entry. Molecular docking studies have indicated that BBR interacts with the HCV E2 glycoprotein [47], suggesting that BBR could be a good candidate for the development of entry inhibitors for the prophylaxis and treatment of HCV infection. The antiviral effect of BBR does not seem to involve modulation of host cell functions such as the interferon response [47–49]. BBR also exhibits antiviral activity against dengue virus (DENV) and Zika virus (ZIKV) infections. Both of these viruses belong to the family *Flaviviridae*. DENV has four serotypes (DENV-1, DENV-2, DENV-3 and DENV-4), which are transmitted to humans by *Aedes aegypti* and *Aedes albopictus* mosquitoes. The virus is responsible for diseases of different severity, including asymptomatic infection, dengue fever, dengue hemorrhagic fever (DHF), and dengue shock syndrome (DSS), which can be fatal [50]. ZIKV infection can cause congenital syndromes including microcephaly, spasticity craniofacial disproportion, irritability, seizures, and other brain dysfunctions [51]. The non-structural viral proteins NS5 and NS3 are crucial for the replication of the DENV and ZIKV genome. In an *in silico* study by Srivastava, BBR was docked with NS5 methyltransferase of DENV and the NS3 protein of ZIKV using the AutoDock 4.2 tool [52]. The results suggested that BBR might be a novel inhibitor of the non-structural proteins (NS5 and NS3) of DENV and ZIKV with potential to prevent infection. However, this needs to be studied experimentally.

### Activity of BBR against viral-borne respiratory syndromes

BBR is also a possible remedy for infection with severe acute respiratory syndrome coronavirus (SARS-CoV), the etiological agent of the respiratory disease SARS. SARS-CoV belongs to the family *Coronaviridae*. Recently, coronaviruses have drawn much attention due to the current pandemic, which has life-threatening health consequences. SARS-CoV is an enveloped, single-stranded, positive-sense RNA with a genome of 29.7 kbp. During infection, the SARS-CoV proteins nsp1, nsp2, nsp7, spike, and nucleocapsid promote NF-κB activation. NF-κB is a DNA-binding protein that regulates the transcription of different genes whose products are involved in the control of cellular processes such as inflammatory and immune responses. During SARS-CoV infection, the virus regulates the expression of pro-inflammatory mediators such as TNF, CCL2, and CXCL2. BBR has an inhibitory effect on the NF-κB signaling pathway and therefore might function as an antiviral agent against coronavirus infection [53, 54]. Recent studies have indicated that an extract from *Coptidis rhizoma* containing BBR and other protoberberine alkaloids might inhibit coronavirus RNA synthesis and viral assembly and release [55]. It might be worth investigating the potential of BBR against SARS-CoV-2 in future studies.

Shinae et al. showed that the replication of RSV was significantly reduced by treatment with BBR [56]. RSV is a member of the family *Paramyxoviridae* and has a negative-sense, nonsegmented RNA genome. RSV infects the respiratory tract of most children before their second birthday and is a common cause of bronchiolitis and pneumonia in infants under the age of 1 year [57]. It has also been recognized as a significant cause of respiratory illness in older adults. During RSV infection, phosphorylation of p38 MAPK occurs at a very early stage of virus replication, and this phosphorylation can be reduced by BBR treatment. The precise molecular mechanism of this inhibition is still unknown, but recent studies have demonstrated that the effect of BBR is based on its direct interaction with a component of the TLR4 receptor complex. BBR can inhibit TLR4 activation and thus suppress p38 MAPK activation.

In addition, the production of interleukin 6 (IL-6) mRNA upon RSV infection is suppressed by BBR, which indicates its anti-inflammatory role during RSV infection [58]. It has been shown that BBR functions through several pathways, such as the NF-κB, ERK1/2 and p38 MAPK. As a consequence, these functions decrease the levels of several proinflammatory cytokines, including tumor necrosis factor alpha (TNF-α), interleukin-1 beta (Il-1β), interleukin-6 (IL-6), and prostaglandin E2 (PGE2). BBR also inhibits the phosphorylation of NF-κB and IκBα) [59].

### Anti-influenza activity

Although influenza virus often causes only mild respiratory illness, influenza can be a life-threatening infectious disease, especially in children, seniors, and immunocompromised patients. Yan et al. observed an antiviral effect of BBR against influenza H1N1 virus [60]. In some countries, seasonal influenza affects up to 40% of the population annually, and worldwide, up to 500 million people die from it each year [61, 62]. Influenza viruses (types A, B, C) are segmented, negative-strand RNA viruses belonging to the family *Orthomyxoviridae*. Influenza A viruses of various subtypes infect many animal species (e.g., birds, swine, horses, dogs, marine mammals, and felids) as well as humans. Influenza A viruses are classified into highly pathogenic subtypes based on their surface glycoproteins, with 17 hemagglutinin (HA) and 10 neuraminidase (NA) subtypes currently recognized. The neuraminidase and hemagglutinin proteins dominate the virion surfaces and are responsible for virus infectivity. NA plays a role in virus replication by releasing new virus particles from host cells, separating them from the neuraminic-acid-containing glycan structures on the surface of the infected cell.

Yan et al. showed that BBR inhibits influenza virus replication in human pulmonary adenocarcinoma cells (cell line A549) and mouse lungs by suppressing the infection. This research confirmed that BBR inhibits the expression of TLR7 and NF-κB, both of which are upregulated in influenza-infected lung tissues [63]. The infection is recognized by host pattern-recognition receptors (PRRs), for example, Toll-like receptors (TLRs), whose signaling pathways converge on two families of transcription factors: NF-κB and interferon regulatory factor (IRF). Both of these factors are translocated to the nucleus, where they upregulate proinflammatory and antiviral responses [64]. Kim et al. showed that extracts from Cortex Phellodendri enriched in BBR can regulate the antiviral host response. It has been shown that BBR modulates the generation of proinflammatory substances such as cytokines and stimulates the antiviral state in infected host cells. The potential therapeutic mechanism of BBR in influenza-associated viral pneumonia might be the result of both inhibition of the viral infection and modulation of the release of inflammatory factors [65, 66]. Other studies have shown that BBR and its derivatives also inhibit cytopathogenic effects and neuraminidase (NA) activity *in vitro.* Enkhtaivan et al. showed that the active site of the viral NA can be blocked by berberine derivatives (BDs) (Table 1) in the same way as it is blocked by the antiviral drug oseltamivir (a well-known NA inhibitor) [65]. The inhibition of viral NA was confirmed in a molecular docking study using BD and the neuraminidases of both influenza A and B viruses [67].

**Table 1:**
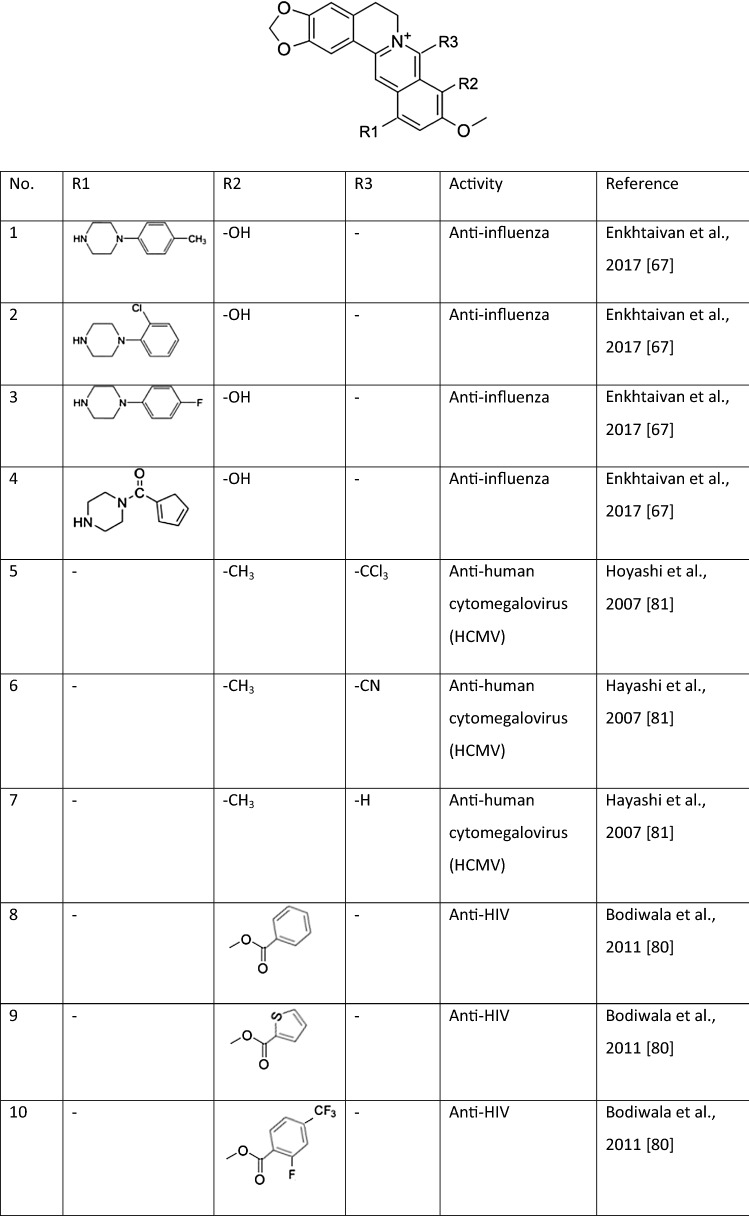
Examples of berberine derivatives with antiviral activity

Using this example of the anti-influenza activity of BBR and its derivatives, we can see that BBR is multifunctional and acts through diverse mechanisms. It can attach to protein molecules at their active sites, hence directly blocking their activity, as is the case with the influenza virus NA protein [67], and it can activate different signaling pathways leading to antiviral activity, e.g., inhibition of the expression of TLR7 and NF-κB [64]. The regulatory effects of BBR on the TLR signaling pathway has also been shown to affect the process of intestinal mucosal damage in rats, but the exact molecular interactions between BBR and other molecules remain to be identified. However, based on the existing evidence, we might assume that they rely on binding of BBR to their active sites [68].

### Anti-inflammatory properties of BBR

BBR has also anti-inflammatory properties and is able to inhibit inflammatory cell infiltration and the production of TNF-α, IL-13, Il-6, IL-8 and IFN-γ [69]. This may occur through the activation of AMP-activated protein kinase (AMPK) and inhibition of NF-κB. It is noteworthy that in some viral infections, e.g., human cytomegalovirus (HCMV) and Ebola virus, AMPK activation has an adverse effect. The mechanism of the anti-inflammatory effect of BBR is complex. BBR can inhibit the binding activity NF-κB and activator protein 1 (AP1). AP1 and NF-κB are key transcription factors that are responsible for regulating the expression of many genes involved in inflammation [69]. Moreover, the anti-inflammatory properties of BBR also involve the modulation of MAPKs. BBR can inhibit generation of proinflammatory cytokines and moderate the inflammatory response. During RSV infection, BBR decreases interleukine-6 (IL-6) mRNA level. In influenza virus infection, BBR reduces the mRNA expression of TLR7 and NF-κB in lung tissue [70].

Inflammation is an important aspect of the pathogenesis of Venezuelan equine encephalitis virus (VEEV) and is associated with the upregulation of multiple mediators, such as TLR signaling, cytokines, inducible nitric oxide synthase (iNOS), TNF-α, TGF-β, interleukins, and chemokines [65]. VEEV is a member of the genus *Alphavirus*, family *Togaviridae*, and has a positive-sense RNA genome. This virus causes severe encephalitis in humans. Four antigenic varieties of VEEV are known, namely IA/B, IC, ID and IE. Three of them, subtypes IA, IB and C are the epizootic strains that cause disease and lead to high mortality in equines. VEEV infection in humans is asymptomatic during the first 1-5 days, followed by the onset of a febrile illness that is characterized by fever, vomiting, headaches, myalgia, ocular pain, or diarrhea, which can last for 1-4 days. The disease can then progress to severe neurological disease, with an incidence of almost 14% [72]. Recent research suggests that BBR might be a potential therapeutic agent against VEEV [65].

### Anti-HPV effect

BBR also suppresses human papillomavirus (HPV) transcription. HPVs are non-enveloped, epitheliotrophic viruses with a circular double-stranded DNA genome that belong to the family *Papillomaviridae*. Persistent infection with high-risk HPVs, such as HPV16 and HPV18, can lead to the development of cervical cancer. Cancer development and progression are driven by the expression of two oncogenes, E6 and E7. Their expression is mainly dependent on the viral E2 protein and on the availability of the host transcription factor activator protein 1 (AP1). HPV E6 and E7 interact with tumor suppressor proteins, p53 and Rb, respectively. E6 binds and induces ubiquitin-mediated degradation of p53, while E7 inactivates the Rb protein and alters additional cellular signaling pathways that are important for transformation. BBR can effectively target both the host AP1 and the viral oncoproteins E6 and E7. Inhibition of AP1 and blocking of viral E6 and E7 oncoprotein expression seem to be among the anti-HPV mechanisms of action of BBR [73].

### Activity of BBR against members of the families *Herpesviridae* and *Picornaviridae*

Low (micromolar) concentrations of BBR can also suppress the replication of different HCMV strains that are resistant to known DNA polymerase inhibitors. HCMV is a member of the family *Herpesviridae* and has a dsDNA genome. HCMV is responsible for life-threatening pneumonia, gastrointestinal diseases, retinitis, and other conditions after primary infection and in immunocompromised patients. It also induces congenital defects in newborn infants, causing neurological disorders in approximately 0.1% of congenital infections. Lunganini et al. showed that BBR interferes with the transactivating function of the HCMV IE2 protein. IE2 plays a critical role in the progression of HCMV replication and in viral pathogenesis and reactivation from latency [74]. It is the most important HCMV regulatory protein and a strong transcriptional activator of viral and cellular gene expression. IE2 binds to DNA and has the ability to interact with cellular transcription factors, which is necessary for regulation of transcriptional activation of viral and host genes and cellular functions [75].

The inhibitory activity of BBR on the IE2-dependent transactivation of early genes depends on activation of MAPKs. BBR is active against human herpes simplex virus types 1 and 2 (HSV 1 and 2) and also against mouse cytomegalovirus (MCMV). HSV infection is characterized by small blisters on the skin or mucous membranes of the mouth, often called “cold sores” or “fever blisters”, and can cause a sore throat. It has been reported that BBR inhibits DNA synthesis by intercalating into DNA. BBR also inhibits the synthesis of both HSV-1 and HSV-2 late genes and proteins [76]. Inhibitory activity of BBR has also been observed against different genotypes of enterovirus 71 (EV71), which belongs to the family *Picornaviridae* and has a positive-sense RNA genome. This virus is the primary cause of hand, foot, and mouth disease, which spreads among infants and young children. Wang et al. showed that BBR and its derivatives (including a variety of esters and ethers at positions 3 and 9) (Table 1) exert moderate activity against EV71 replication. This is achieved mainly through downregulation of MEK/ERK signaling, inhibition of EV71-associated autophagy by activation of AKT, and suppression of the phosphorylation of JNK [77].

### Anti-HIV activity

Human immunodeficiency virus type 1 (HIV-1) is the causative agent of the worldwide acquired immunodeficiency syndrome (AIDS) epidemic. Approximately 38 million people were estimated to live with HIV in 2018. Acute HIV infection often manifests clinically as a nonspecific viral infection syndrome with sore throat, fever, lymphadenopathy, or aseptic meningitis. HIV belongs to the family *Retroviridae*, subfamily *Orthoretrovirinae*.

The HIV genome consists of two identical single-stranded RNA molecules that are enclosed within the core of the virus particle. The virus has a very high genetic variability. The genome of the HIV provirus, also known as proviral DNA, is generated by reverse transcription of the viral RNA genome into DNA, degradation of the RNA, and integration of the double-stranded HIV DNA into the host genome. HIV is a retrovirus that occurs as two types: HIV-1 and HIV-2. HIV protease is an important enzyme for viral maturation. It cleaves Gag and the Gag-Pol polyprotein precursor at nine sites to produce mature active proteins. Therefore, HIV protease inhibitors (PIs) are used in highly active antiretroviral therapy (HAART) against HIV infection. However, some inhibitors induce expression of TNF-α and IL6, which are major mediators of the inflammatory response and are implicated in the pathogenesis of the variety of inflammatory diseases, including atherosclerosis [78]. BBR significantly inhibits HIV-PI-induced TNF-α and IL6 expression and ERK signaling [79].

BBR and berberrubine along with 9-substituted derivatives of berberine have demonstrated antiviral activity against HIV [80], probably due to inhibition of reverse transcriptase (RT) activity. The use of 20 µg of BBR per reaction resulted in 94% inhibition of HIV-1 RT. An improved therapeutic effect was observed when berberine-*9-0* esters were used [80] (Table 1). BBR might therefore have a potential application as a complimentary therapeutic agent against HIV infection.

## Conclusions

Recent studies have demonstrated therapeutic activity of BBR and its derivatives, especially against viral entry and replication. BBR has the ability to inhibit infection of various viruses including influenza virus, HSV, HCMV and CHIKV, and to reduce virus production. For some of these viral infections (e.g. CHIKV) there are still no approved drugs or treatments. Many viruses can target the MAPK pathway to manipulate cellular functions and control viral replication, leading to host cell death. These pathways are also involved in inhibitory effect of BBR. In addition, BBR can inhibit inflammatory responses triggered by viruses. Interestingly, in recent years, many scientific reports have reported immunostimulating and anti-inflammatory activity of BBR. Recent research suggests that BBR and its derivatives are active plant biomolecules that can be applied successfully for antiviral pharmacological strategies, possibly and hopefully also against SARS-CoV-2, which is currently a major problem worldwide.
